# An In Vitro Partial Lesion Model of Differentiated Human Mesencephalic Neurons: Effect of Pericyte Secretome on Phenotypic Markers

**DOI:** 10.1007/s12031-020-01589-6

**Published:** 2020-05-29

**Authors:** Abderahim Gaceb, Marco Barbariga, Gesine Paul

**Affiliations:** 1 Translational Neurology Group, Department of Clinical Science, Wallenberg Neuroscience Center and Wallenberg Center for Molecular Medicine, Lund University, Sölvegatan 19, 22184 Lund, Sweden; 2 Department of Neurology, Scania University Hospital, 22185 Lund, Sweden

**Keywords:** Brain pericytes, Dopaminergic neurons, Partial lesion, Secretome, Platelet-derived growth factor BB, Neurorestoration

## Abstract

Parkinson’s disease (PD) is characterised by the progressive degeneration of dopaminergic (DA) neurons in the substantia nigra pars compacta. Post-mortem data suggests that the loss of DA markers may long precede the cell death, leaving a window to rescue the DA phenotype. Screening for potential neuroprotective or restorative therapies, however, requires that partial lesions of DA neurons can be modelled in vitro. In order to establish a partial lesion model of DA neurons in vitro, we evaluated the effects of different exposure times to 1-methyl-4-phenylpyridinium (MPP^+^) and 6-hydroxydopamine (6-OHDA) on the cell survival and DA marker expression using DA neurons derived from the Lund human mesencephalic (LUHMES) cell line. We show that 24-h incubation with 50 μM of MPP^+^ or 6-h incubation with 100 μM of 6-OHDA leads to a significant decrease in the protein expression of DA markers without affecting overall cell death, consistent with a mild DA lesion. Using conditioned medium of human brain–derived pericytes stimulated with platelet-derived growth factor BB (PDGF-BB), we demonstrate a significant upregulation of DA markers. In conclusion, we provide an experimental model of an in vitro DA neuron partial lesion suitable to study different molecules and their potential neuroprotective or neurorestorative effects on the DA phenotype. We provide evidence that the secretome of brain pericytes stimulated via PDGF-BB/PDGFRβ affects DA marker expression and may represent one possible mechanism contributing to the neurorestoration previously observed in PD by this growth factor.

**Electronic supplementary material:**

The online version of this article (10.1007/s12031-020-01589-6) contains supplementary material, which is available to authorized users.

## Introduction

Parkinson’s disease (PD) is a slowly progressive neurodegenerative disease characterised by the continuous and progressive degeneration of several neuronal subtypes (Jellinger 1991). The most affected cells are the dopaminergic (DA) neurons in the substantia nigra pars compacta (SNpc), a cell type that has become the primary target for neuroprotective and neurorestorative approaches.

The cause of DA neuron death is still debated and may depend on impaired protein degradation systems, synaptic dysfunction, endoplasmic reticulum stress, DNA damage, mitochondrial dysfunction, and inflammation (Seward et al. 2013). Interestingly, post-mortem data suggest that the loss of melanised neurons is long preceded by the loss of DA markers suggesting a possible downregulation of phenotypic markers in dopaminergic neurons (Kordower et al. 2013) leaving DA neurons impaired but still viable. Importantly, this downregulation of the DA phenotype may leave a time window to rescue and restore the DA phenotype using neuroprotective or neurorestorative interventions. To investigate such potential restorative therapies, partial lesions of DA neurons need to be modelled where the marker expression characteristic of the DA phenotype is compromised but can be rescued.

DA neurons are characterised by the expression of the enzyme tyrosine hydroxylase (TH), a catecholamine synthesis enzyme, dopamine transporter (DAT), dopamine receptor D2 (DRD2), dopamine beta-hydroxylase (Dopa-β-H), vesicular monoamine transporter 2 (VMAT2), and many other neuronal markers (Lindh and Hokfelt 1990). Lund human mesencephalic (LUHMES) cells are immortalised human foetal neuronal precursor cells that have been extensively used in in vitro models of neurodegeneration (Lotharius et al. 2002; Lotharius et al. 2005; Smirnova et al. 2016). LUHMES were originally immortalised from 8-week-old human foetal ventral mesencephalic cells with a tetracycline-regulated v-myc expression vector (Hoshimaru et al. 1996), which allows a proliferation arrest and the neuronal differentiation with the addition of tetracycline to the medium. LUHMES cells can be maintained as proliferating cells or can be differentiated to postmitotic neurons by the addition of tetracycline, glial cell–derived neurotrophic factor (GDNF), and dibutyryl cyclic AMP (db-cAMP) (Hoshimaru et al. 1996; Schildknecht et al. 2009), which allows differentiation towards a DA phenotype. The properties of these cells can be considered to be as similar to adult human DA neurons as possible.

Toxins such as 1-methyl-4-phenylpyridinium (MPP^+^) and 6-hydroxydopamine (6-OHDA) are widely used to model PD in vivo and in vitro (Bove and Perier 2012; Kowall et al. 2000; Presgraves et al. 2004; Salari and Bagheri 2019; Zeng et al. 2018). MPP^+^ and 6-OHDA induce DA toxicity by increasing oxidation and mitochondrial dysfunction (Blum et al. 2001; Richardson et al. 2007; Subramaniam and Chesselet 2013). Neuroprotection or neurorestoration of the DA cells is currently one of the major unmet clinical needs in PD. Several growth factors have been explored in preclinical models (Paul and Sullivan 2019). One of the few growth factors that has reached clinical trials is platelet-derived growth factor (PDGF)-BB (Paul and Sullivan 2019; Paul et al. 2015). PDGF-BB has been shown to have a restorative effect on the nigrostriatal system in partial lesion in vivo PD models (Padel et al. 2016; Zachrisson et al. 2011), findings that have led to clinical trials (Paul et al. 2015). However, despite these intriguing findings, how the restorative effect of PDGF-BB on the DA phenotype is mediated is currently not known. Interestingly, the nigrostriatal restoration and behavioural recovery seen in PD models were associated with a normalisation of the vasculature and specifically normalisation of activated pericytes (Padel et al. 2016) suggesting that pericytes are the mediating cell type.

Brain pericytes are known to highly express the receptor beta for PDGF-BB, PDGFRβ. Apart from a variety of other functions, pericytes have extensive secretory abilities and may possess pro-regenerative capacities in response to PDGF-BB (Gaceb et al. 2018; Gaceb and Paul 2018). However, whether the secretome of pericytes stimulated with PDGF-BB has an impact on DA marker expression has so far not been investigated.

The purpose of the present study was to establish an in vitro lesion model suitable to screen different neuroprotective/neurorestorative molecules used in PD therapies by titrating a toxin-induced partial lesion of human DA cells that leads to a compromised DA phenotype without causing overall cell death and to investigate whether PDGF-BB-stimulated pericytes might be the mediating cell type affecting the expression of DA phenotypic markers.

We used the LUHMES cell line to evaluate the effects of short-term and long-term exposure to MPP^+^ or 6-OHDA on cell survival and DA markers expression and identified experimental conditions consistent with an in vitro model of a partial lesion of DA neurons suitable to screen neuroprotective/neurorestorative molecules. Furthermore, using conditioned medium from human PDGF-BB-stimulated pericytes, we provide evidence that the secretome of brain pericytes modulates the phenotypic marker expression of human DA neurons in vitro.

## Materials and Methods

### LUHMES Differentiation

LUHMES cells were grown and differentiated as described before (Lotharius et al. 2002; Lotharius et al. 2005). Briefly, cells were propagated in pre-coated (Poly-d-lysine 5 μg/ml, mouse laminin 5 μg/ml O/N) flasks in advanced Dulbecco’s Modified Eagle Medium (DMEM)/F-12 high-glucose medium added with N2 supplement (100 ×) (Invitrogen, Glostrup, Denmark), L-glutamine (200 mM) (Sigma-Aldrich), and 160 μg/ml basic fibroblast growth factor (bFGF) (R&D Systems). For the differentiation step, cells were seeded in a T75 pre-coated flask (3 × 10^6^ cells) and let to grow for 24 h in proliferation medium. The medium was then replaced with differentiation medium composed of advanced DMEM/F-12 high-glucose medium containing N2 supplement (100 ×), L-glutamine (200 mM), 1 mg/ml tetracycline, 10 mM db-cAMP (Sigma-Aldrich), and 20 μg/ml GDNF (R&D Systems, Minneapolis, MN) for 48 h. LUHMES cells were then enzymatically dissociated with trypsin and seeded in pre-coated multi-well plates at a density of 1 × 10^6^ cells/well in 6-well plates, or 1 × 10^4^ cells/well in 96-well plates depending on the assay conducted (western blot/qPCR and MTT (3-(4,5-diméthylthiazol-2-yl)-2,5-diphényltétrazolium) viability assay, respectively). Nine independent experiments in triplicates were performed for western blot/qPCR and the MTT viability assay. Cells were let to differentiate in differentiation medium for 5 days, replacing half of the medium every other day.

### LUHMES Lesion

After 5 days of incubation with differentiation medium, LUHMES cells showed the morphology of mature neurons as observed with phase-contrast microscopy. Differentiated LUHMES cells were treated on day 6 with either MPP^+^ (1-methyl-4-phenylpyridinium, Sigma-Aldrich) or 6-OHDA (Sigma-Aldrich). Toxins were dissolved in 0.02% ascorbate/phosphate-buffered saline (PBS) solution at the concentration of 5 mM and added to fresh differentiation medium to obtain three different final concentrations of 5, 15, 50 μM MPP^+^ or 25, 50, 100 μM 6-OHDA, respectively. For the control experiments, cells were in the media without toxins or vehicle added. Cultures were incubated with the respective toxins in the above concentrations for two different time lengths, 6 or 24 h, respectively.

### Human Brain Vascular Pericytes

Human brain pericytes (ScienCell Research Laboratories, Carlsbad, CA, USA) were plated and expanded in Stemline medium (Sigma-Aldrich) supplemented with 2% foetal bovine serum (Invitrogen), 1% penicillin/streptomycin (Gibco), 20 ng/ml bFGF (Invitrogen) on gelatin-coated culture flasks (Nunc), and incubated at 37 °C in 5% CO_2_ conditions (Heraeus HERAcell 150 CO_2_ incubator, Thermo Scientific). Cells grew exponentially with a doubling time of approximately 48 h, reaching ca. 85% confluence after 48–72 h. The cells were seeded in six-well culture plates at 100,000 cells/well for the following experiments. The cells were expanded and used between passages 2–5. As previously described, human brain pericyte–conditioned medium was collected from non-treated pericyte-conditioned medium (CM) and pericytes treated with PDGF-BB (20 ng/ml, RD systems) (CM^PDGFBB^) for 72 h (Gaceb et al. 2018). Briefly, cell medium was centrifuged at 1500*g* for 5 min, and the supernatant free from cell debris was collected and used for the following experiments. At 72 h, human brain pericyte–conditioned medium shows only a very low contamination with the added PDGF-BB (Gaceb et al. 2018).

### Flow Cytometry

Human brain pericytes (passage 2, three independent experiments in triplicates) were harvested (20,000 cells) and washed three times with PBS. Cells were incubated with anti-human CD140b-PE (#558821), anti-human CD31-FITC (#560984), (BD Biosciences, San Jose, CA), and anti-human CD146-FITC (#MCA2141F) (AbD Serotec) fluorochrome-conjugated antibodies, in separated tubes for 45 min at 37 °C in the dark. IgG1-PE (#555574) and FITC (#555748) mouse antibodies (BD Biosciences) were used as isotype control to set the threshold and gates in the cytometer. Finally, samples were analysed by flow cytometry with an Accuri C6 (BD Biosciences). Briefly, the region corresponding to pericytes was defined by a combination of forward (FSC) and side scatter (SSC) properties. Isotype control and unstained pericytes were used to set the background and the gate settings. Pericytes were identified according to the expression of membrane-specific antigens (CD140b, CD146, CD31) in the PE and FITC fluorescence channels. A total of 500,000–1,000,000 events were recorded for each sample and analysed with the FACS Diva software (Tree Star, Inc.).

### qPCR

Following experimental treatment, total RNA was purified from LUHMES cells with micro-RNAeasy RNA (Qiagen) according to the manufacturer’s instructions. Total RNA specimens were treated with DNase to remove contaminated genomic DNA. RNA purity and concentration were determined using the NanoDrop 2000C spectrophotometer (Thermo Scientific), and specimens were subsequently stored at − 80 °C before use. The first-strand cDNA of the total extracted RNA obtained from each specimen was synthesised according to instructions from the manufacturer using the iScript cDNA Synthesis Kit (Bio-Rad). Gene expression was then assessed with the SYBR® Green Master Mix kit (Bio-Rad) according to the manufacturer instructions and analysed with CFX96 Touch™ Real-Time PCR Detection System. All values were normalised to GAPDH gene expression (housekeeping gene). Constant GAPDH gene expression was confirmed experimentally between proliferation and differentiation LUHMES. Primers for TH, DAT, synaptophysin (SYP), synapsin I (SYN), dopamine receptor D2 (DRD2), and neuron-specific class III beta-tubulin (TUJ1) were used (see Table 1 for primer sequences).

**Table 1:** Primer sequences for polymerase chain reaction

	Primer sequence
Tyrosine hydroxylase (TH)	Right: GGTGGATTTTGGCTTCAAAC
	Left: CTGTGGCCTTTGAGGAGAAG
Dopamine transporter (DAT)	Right: TGCTCCGTGATGTAGCGGTT
	Left: CACCATCTTCCAGGAGCGAGATC
Synaptophysin (SYP)	Right: CGAGGTCGAGTTCGAGTACC
	Left: AATTCGGCTGACGAGGAGTA
Synapsin I (SYN)	Right: TCAGACCTTCTACCCCAATCA
	Left: GTCCTGGAAGTCATGCTGGT
Dopamine receptor D2 (DRD2)	Right: GCAGGAGGCATTGCTGATGAT
	Left: TGGAGGATTCCCCATATGAA
Neuron-specific class III beta-tubulin (TUJ1)	Right: CGCCCAGTATGAGGGAGAT
	Left: AGTCGCCCACGTAGTTGC

### MTT Viability Assay

To determine cell viability, 3-(4,5-dimethyl-2-thiazolyl)-2,5-diphenyl-2 H-tetrazolium bromide (MTT) 5 mg/ml was added to the medium at the end of each experiment for 2 h at 37 °C. The medium was then removed, and cells were lysed in 100 μl dimethyl sulfoxide (DMSO) (Sigma-Aldrich) for 10 min. Optical density was read at 595 nm using a plate reader (Brand). In addition, live cell counting using trypan blue was performed to evaluate cell viability (see Supplementary Figure 1).

### Protein Extraction and Western Blot

For western blot analysis, differentiated LUHMES were washed three times with PBS and lysed with RIPA buffer (Thermo Scientific, 100 μl each well) supplemented with protease inhibitors (Thermo Scientific). After incubation for 30 min on ice and centrifugation at 15,000 RCF (15 min at 4 °C) to eliminate cell debris, protein concentrations were evaluated with the BCA kit (Matsui et al.). For western blot analysis, 10 μg of each sample were incubated in Laemmli buffer (5 min at 100 °C) and samples were resolved by SDS-PAGE and analysed using rabbit anti-TH antibody (#AB152) (1:500, Millipore), rabbit anti-dopamine beta-hydroxylase antibody (Dopa-β-H) (#ab96615) (1:1000, Abcam), rabbit anti-vesicular monoamine transporter 2 (VMAT2) (#ab87589) (1:1000, Abcam), and anti-β-actin-HRP antibody (#A3854) (1:10,000, Sigma). Images were acquired using the ChemiDoc MP system (Bio-Rad).

### Statistical Analysis

Data were analysed using GraphPad Prism Software and are expressed as mean ± standard deviation (SD). For group comparison, a one-way or two-way ANOVA with a Tukey’s post hoc was used. For two-group comparison, an unpaired two-tailed Student’s *t* test was used. The *p* value was corrected for multiple comparisons, and comparisons were considered significant with a *p* value < 0.05.

## Results

### Differentiation of LUHMES Cells to DA-Like Neurons

**Fig. 1:**
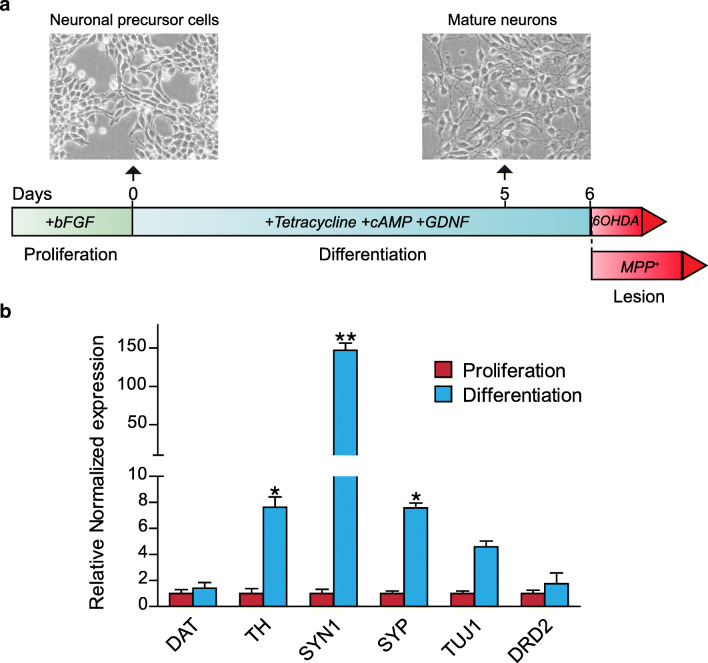
LUHMES differentiation and lesion protocol outline. (A) Differentiation protocol of LUHMES cells and toxin lesion. (B) LUHMES gene expression analysis before and after the differentiation step measured by qPCR. From left to right: dopamine transporter (DAT), tyrosine hydroxylase (TH), synapsin 1 (SYN1), synaptophysin (SYP), class III beta-tubulin (TUJ1), and dopamine receptor D2 (DRD2). The results were normalised and represent the mean **±** SD (9 independent experiments, 3 replicates). Statistical significance reported as *p* values is evaluated by two-way ANOVA with Tukey’s post hoc: *p **<** 0.05, **p < 0.01 vs proliferation group

First, we confirmed the DA nature of LUHMES cells after differentiation. LUHMES cells were grown in proliferation medium for 24 h, which was then replaced with differentiation medium (Fig. 1A). At day 5 of differentiation, the neuronal precursor cells changed their morphology and acquired a mature neuronal morphology with a clearly demarcated cell body under phase contrast and extensive neurites (Fig. 1A). To confirm the DA phenotype of the differentiated LUHMES cells, we analysed the DA gene expression before and after the differentiation step. Differentiated LUHMES cells showed a significant increase in gene expression of neuronal (SYN1, SYP) and DA neuron–specific markers (TH). These results confirmed that our experimental differentiation protocol results in DA-like neurons. After the differentiation step, LUHMES cells were treated on day 6 with MPP^+^ or 6-OHDA to induce the lesion, following the protocol described in Fig. 1A.

### Titration of MPP^+^ and 6-OHDA for Partial Lesion of LUHMES DA Neurons

In order to establish an in vitro partial lesion model where impairment of the DA phenotype without cell death is achieved, we explored the effect of two different toxins known to affect the DA phenotype: MPP^+^ and 6-OHDA. We tested the impact of different concentrations and exposure times on cell morphology, cell viability, and expression of several DA-related phenotypic markers.

Based on previous studies, we used the following concentrations 5, 15, and 50 μM for MPP^+^ and 25, 50, and 100 μM for 6-OHDA and exposed cells for two different time lengths, 6 or 24 h, respectively (Lotharius et al. 2005; Schildknecht et al. 2009). No differences were observed between the MTT assay and trypan blue cell counting (see Supplementary Figure 1).

**Fig. 2:**
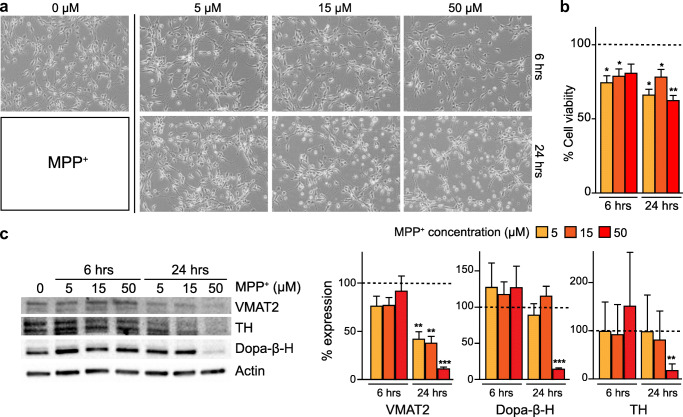
MPP^+^ toxin titration. (A) Morphology of differentiated LUHMES cells after in vitro lesion with different concentrations of MPP^+^ for 6–24 h. (B) LUHMES cell viability after MPP^+^ lesion, normalised on non-lesioned cells, and measured with MTT assay. **c** Western blot analysis and corresponding quantifications of dopamine metabolism–related proteins; vesicular monoamine transporter 2 (VMAT2), dopamine beta-hydroxylase (Dopa-β-H), and tyrosine hydroxylase (TH) protein expression. The results were normalised to non-lesioned cells (9 independent experiments, 3 replicates). Statistical significance reported as *p* values is evaluated by Student’s *t* test. ***p < 0.001, **p < 0.01, *p < 0.05

A 24-h exposure of differentiated LUHMES to MPP^+^ in a concentration of 50 μM led to morphological changes (Fig. 2A) and a significant decrease in protein expression of markers related to DA metabolism as TH, VMAT2, and Dopa-β-H (Fig. 2C), whereas cell survival, measured with the MTT assay, was only reduced by 25%, indicating a mild lesion that did not cause overall cell death (Fig. 2B). However, 24-h incubation with 5 and 15 μM of MPP^+^ decreased only the expression of VMAT2 but not TH and Dopa-β-H (Fig. 2C). MPP^+^ exposure at concentrations of 5, 15, and 50 μM for 6 h had similar effects on cell morphology and viability but did not affect the protein expression of DA markers (Fig. 2A–C).

**Fig. 3:**
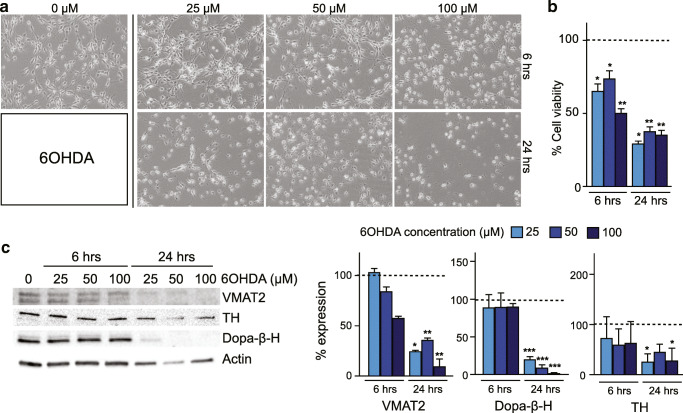
6-OHDA toxin titration. (A) Morphology of differentiated LUHMES cells after in vitro lesion with different concentrations of 6-OHDA for 6–24 h. (B) LUHMES cell viability after 6-OHDA lesion, normalised to non-lesioned cells, and measured with MTT assay. (C) Western blot analysis and corresponding quantifications of dopamine metabolism–related proteins, vesicular monoamine transporter 2 (VMAT2), dopamine beta-hydroxylase (Dopa-β-H), and tyrosine hydroxylase (TH) protein expression. The results were normalised to non-lesioned cells (9 independent experiments, 3 replicates). Statistical significance reported as *p* values is evaluated by Student’s *t* test. ***p < 0.001, **p < 0.01, *p < 0.05

We also investigated 6-OHDA at three different concentrations to see whether it was superior to MPP^+^. Exposure of cultures for 6 h with 100 μM 6-OHDA resulted in morphological changes with a significant decrease in cell viability (50%) and a slight decrease of DA-related markers expression (Fig. 3A–C). Exposure to 25 and 50 μM of 6-OHDA for 6 h led to morphological changes with a significant decrease in cell viability but had no effect on the protein expression of DA markers (Fig. 3A–C). Incubation with 6-OHDA for 24 h resulted in morphological changes, overall cell death (≈ 70%), and a significant decrease in DA markers at all the concentrations tested (Fig. 3A–C).

These data provided the specific experimental conditions to induce a DA partial lesion (24-h incubation with 50 μM of MPP^+^ or 6-h incubation with 100 μM of 6-OHDA), whereby MPP^+^ was more effective to reduce DA markers. We therefore carried these specific experimental conditions of toxin concentration/ time of treatment as a model of a partial lesion forward into the next part of the study.

### Human Brain Vascular Pericytes Highly Express PDGFRβ

We next investigated whether the partial loss of the DA phenotype markers of LUMES cells is reversible.

Recently, we suggested that the brain pericyte secretome mediated via PDGF-BB/PDGFRβ may be a mechanism underlying the restoration of DA phenotype marker observed in PD upon PDGF-BB (Gaceb et al. 2018). We therefore investigated whether the pericyte secretome has an impact on DA phenotypic marker expression in vitro.

**Fig. 4:**
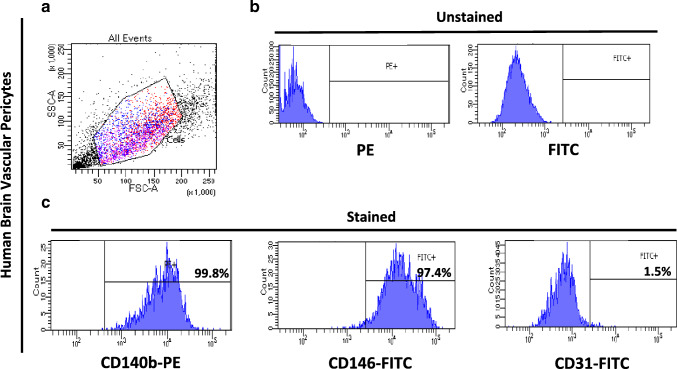
Characterisation of human brain vascular pericytes by flow cytometry. (A) Cells were identified according to forward scatter (FSC) and side scatter (SSC) intensity. (B) Histograms of unstained human brain pericytes. (C) Histograms represent the fluorescence intensity of human brain pericytes after incubation with CD140b, CD146, and CD31 antibodies, respectively. (3 independent experiments, 3 replicates)

We first evaluated the identity and purity of human brain pericytes before PDGF-BB treatment (Guijarro-Munoz et al. 2014; Guijarro-Munoz et al. 2012). Using flow cytometry, cell surface marker expression was analysed for the presence of the pericyte markers PDGFRβ (CD140b) and CD146 and for the absence of the endothelial marker CD31 (Fig. 4A–C). Our results confirmed that PDGFRβ (CD140b) and CD146 were highly expressed on human brain pericytes (99.3% and 97.4%, respectively). As previously shown, these results confirm that these human brain pericytes are a highly homogenous population and express pericyte markers (Guijarro-Munoz et al. 2014).

### Pericyte-Conditioned Medium Restores the DA Phenotype After MPP^+^ Partial Lesion

**Fig. 5:**
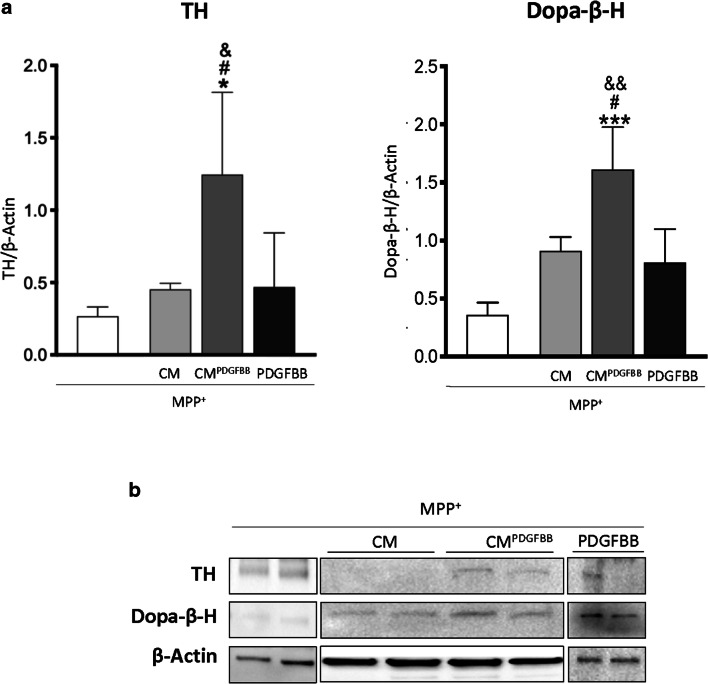
Effect of pericyte-conditioned medium on DA phenotype restoration. Western blot analysis and corresponding quantifications of tyrosine hydroxylase (TH) and dopamine beta-hydroxylase (Dopa-β-H) protein expression (A, B). Differentiated LUHMES cells were treated with MPP^+^ 50 μM for 24 h and then incubated with non-treated pericyte–conditioned medium (CM); conditioned medium of PDGF-BB treated pericytes (CM^PDGFBB^), or with PDGF-BB alone for 24 h. The results were normalised to β Actin, expressed as fluorescence intensity and represent the mean **±** SD (3–6 independent experiments, 3 replicates). Statistical significance reported as *p* values are evaluated by one-way ANOVA with Tukey’s post hoc: *p **<** 0.05, ***p < 0.001 vs MPP^+^ group; ^#^*p* < 0.05 vs CM group; ^&^*p* < 0.05, ^&&^*p* < 0.01 vs PDGF-BB group

We have previously shown that human brain pericytes secrete a number of pro-regenerative molecules in response to PDGF-BB (Gaceb et al. 2018). To evaluate the effect of conditioned medium of PDGF-BB-treated pericytes on DA phenotype restoration, differentiated LUHMES cells were treated first with MPP^+^ 50 μM for 24 h to establish the partial lesion and then incubated with conditioned medium of PDGF-BB-treated pericytes (CM^PDGFBB^), non-treated pericyte-conditioned medium (CM) or PDGF-BB alone. The conditioned medium of both stimulated and unstimulated pericytes did not affect LUHMES viability (Supplementary Figure 1). Interestingly, western blot analysis showed that conditioned medium of PDGF-BB-treated pericytes (CM^PDGFBB^) significantly increased TH and Dopa-β-H protein expression compared with other treatments (Fig. 5 A and 5B), whereas non-treated pericyte-conditioned medium (CM) and PDGF-BB alone showed only a non-significant trend to increase TH and Dopa-β-H protein expression (Fig. 5 A and B). These data suggest an additional effect of the secretome of pericytes after being stimulated with PDGF-BB on the restoration of DA markers.

## Discussion

**Fig. 6:**
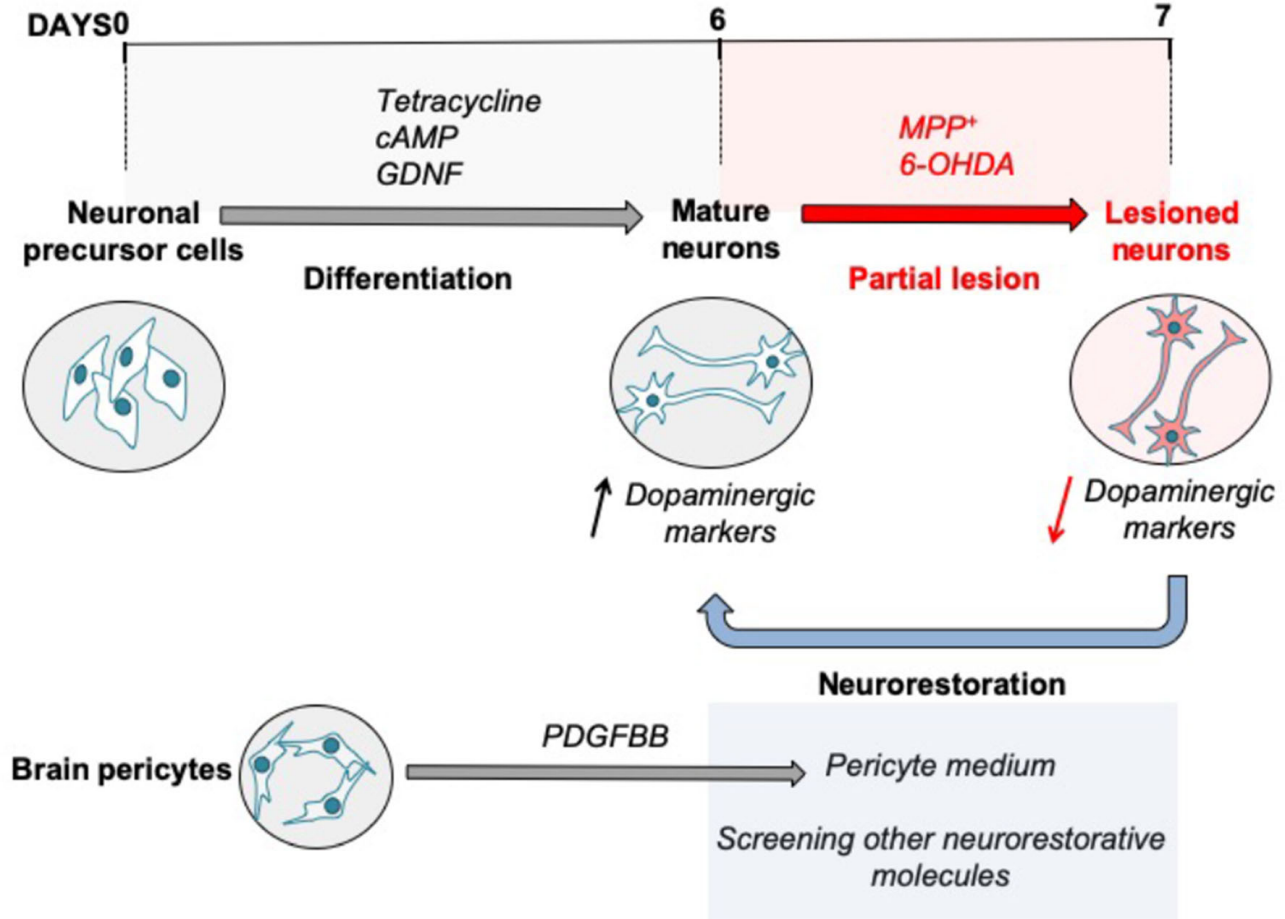
Summary of the proposed experimental strategy. Differentiation of neuronal precursor cells to mature DA neurons is characterised by cell morphology maturation and increase of DA markers. DA cells partial lesion is obtained after incubation with the toxic factors (MPP^+^ or 6-OHDA). The neurorestorative capacities of conditioned medium collected from PDGF-BB-treated pericytes or other target molecules are evaluated by the rescue of the phenotype of lesioned neurons

We have established an in vitro partial lesion model of human DA neurons (LUHMES), investigating different concentrations of MPP^+^ or 6-OHDA (Fig. 6). We demonstrated that 24-h incubation with 50 μM of MPP^+^ was superior as it led to a mild DA lesion characterised by a significant decrease in DA marker protein expression without compromising overall cell death. This in vitro PD model might be suitable to screen different neuroprotective/neurorestorative molecules. Using conditioned medium of PDGF-BB-stimulated pericytes, we showed that the DA phenotype in this model is reversible providing evidence that the secretome of human brain pericytes can rescue an impaired DA phenotype by increasing the expression of DA markers expression. Our data lends support to the hypothesis that brain pericytes may present a new actor in the central nervous system regeneration that is related to their secretory abilities (Gaceb et al. 2018).

Several neurotoxins have been utilised to model PD in vitro and in vivo of which MPP^+^ and 6-OHDA are the most widely used ones (Bove and Perier 2012; Presgraves et al. 2004; Salari and Bagheri 2019; Zeng et al. 2018). MPP^+^ causes the specific degeneration of DA neurons being actively taken up via DAT (Dauer and Przedborski 2003) and inhibits the complex I activity inducing mitochondrial dysfunction. This leads to the production of reactive oxygen species (Oschatz et al. 2004) with subsequent oxidative stress (Richardson et al. 2007; Subramaniam and Chesselet 2013). Like MPP^+^, 6-OHDA is taken up by DA neurons via DAT and it is thought to induce DA toxicity by increased oxidation and mitochondrial respiratory chain inhibition (Blum et al. 2001).

Here, we described a partial DA neuron lesion using both MPP^+^ and 6-OHDA neurotoxicity, whereby we defined partial lesion as loss of DA markers without neuronal death. We demonstrate that the neurotoxic effect is both time and concentration dependent and identify experimental parameters that result in a significant decrease in DA markers protein expression without overall cell death after long- or short-term incubation (50 μM MPP^+^ 24 h; 100 μM of 6-OHDA 6 h). The mechanisms underlying the partial lesion are likely related to the cellular bioenergetic disturbance. Previous reports have shown a MPP^+^ dose-dependent reduction in ATP levels with an IC_50_ value of 65 μmol/l and a significant increase of caspase 3/7 activity at 80 μmol/l in differentiated human LUHMES cells (Zhang et al. 2014). Moreover, in LUHMES-derived DA neurons, MPP^+^ depletes cellular ATP at a low concentration of 5 μM with a distinct bioenergetic mechanism (Poltl et al. 2012). Consistent with our findings, 6-OHDA has been shown to decrease the viability of two neuronal cell lines mouse Neuro-2a and human SH-SY5Y cells in a concentration-dependent manner with similar EC50 values (~ 110 μM) 24 h after treatment (Kang et al. 2017).

Several cell culture systems have been employed in order to study the disease process and investigate potential therapies. Here, we established an in vitro disease model using differentiated LUHMES, providing an immortalised homogenous cell population characterised by high proliferation and rapid differentiation. Being a human-derived cell line, LUHMES cells are biologically highly relevant for preclinical research and already extensively used as a neurodegeneration model (Hoshimaru et al. 1996; Lotharius et al. 2002; Schildknecht et al. 2009). Many differentiation protocols now exist for the production of different types of neurons in vitro, using foetal neural stem cells, inducible pluripotent stem cells, or reprogramming adult somatic cells. The majority of these protocols require long differentiation periods often resulting in a low rate of DA cells (Yan et al. 2005) (Zhang et al. 2009). There is still a need to develop and improve in vitro models of DA neurons (Harischandra et al. 2019).

Our partial DA lesion model opens the possibilities to perform a drug screening in vitro using a human cell line. The advantages of this model are many, including (i) usage of human cells instead of rodent cells, (ii) reduction of the use of animal models, (iii) possibility of expansion and replication for several different assays, and (iv) relatively low costs.

In contrast to partial in vitro lesion models, partial lesion PD models in vivo are much more commonly used. We and others have previously characterised an in vivo partial lesion model of PD by injecting 6-OHDA in the middle forebrain bundle of mice, resulting in a ca. 50% nigrostriatal lesion. The degree of lesion is reflected in the behavioural impairments that can be used to evaluate possible future treatments (Boix et al. 2015; Zachrisson et al. 2011). MPP^+^ treatment produces a number of insults to DA neurons seen in mice, rats, and non-human primates (Langston et al. 1984; Lau et al. 1991; Yazdani et al. 2006). MPP^+^ significantly decreased gene expression of TH, DAT, and VMAT, coinciding with DA concentration changes and decreased protein expression in mice models (Xu et al. 2005). These models proved the possibility to study neuroprotective agents in vivo that require remaining DA cells to be rescued and usually show a mild but clearly detectable behavioural impairment.

PDGFs are endogenous growth factors that occur in several different isoforms. The PDGF-B gene product forms the biologically active PDGF-BB dimer, which binds to PDGFRα and β, whereby it has the highest affinity for PDGFRβ (Matsui et al. 1989). Brain pericytes highly express the receptors for PDGF-BB, and PDGF-BB signalling is important for pericyte recruitment to the blood vessels and for vessel stabilisation (Bergers and Song 2005; Gaceb et al. 2018). PDGF-BB has been studied in several partial lesion PD models in vivo (Padel et al. 2016). Intracerebroventricular administration of this growth factor not only leads to behavioural recovery but also was associated with restoration of the nigrostriatal pathway as measured by a significant increase in striatal TH-positive fibre density and a partial restoration of TH-positive cell numbers (Padel et al. 2016). PDGF-BB has also shown to have a partial restorative effect in other animal models of PD including non-human primates (Paul-Visse et al. 2013; Zachrisson et al. 2011). Intracerebroventricular treatment with PDGF-BB in animal models resulted in (a) increased periventricular cell proliferation, (b) an increased number of TH-positive DA neurons in the substantia nigra, (c) partial restoration of striatal DAT levels, and (d) normalisation of Parkinsonian behaviour (Zachrisson et al. 2011). These findings have led to clinical trials investigating PDGF-BB as a possible neurorestorative or neuroprotective compound in patients with PD (Paul and Sullivan 2019; Paul et al. 2015). However, the mechanisms behind PDGF-BB treatment are still unclear. Besides vascular stabilisation, PDGF-BB elicits the secretion of several neuroprotective and angiogenic growth factors by brain pericytes (Gaceb et al. 2018) which may represent a possible mechanism for the restoration of the DA phenotype.

Here, we demonstrate for the first time, an additional effect of the secretome of pericytes on the restoration of DA markers compared with PDGF-BB treatment alone. Brain pericytes have extensive secretory abilities (Gaceb and Paul 2018) and may possess pro-regenerative capacities in response to PDGF-BB (Gaceb et al. 2018). Our data support the hypothesis that the brain pericytes secretome mediated via PDGF-BB/PDGFRβ signalling may present a new actor in central nervous system regeneration. The secretion of neuroprotective molecules by pericytes in response to PDGF-BB might contribute to the restorative effects on the DA phenotype seen in animal models of PD or PD patients treated with PDGF-BB by modulating DA marker expression. PDGFR-β signalling has been shown to be essential for neurovascular functions, and disturbance causes abnormal BBB functions (Armulik et al. 2010; Bell et al. 2010; Daneman et al. 2010). As the neurorestorative mechanism of PDGF-BB is not fully understood, our results lend further support to the hypothesis that the secretome of pericytes in response to PDGF-BB is of high relevance, as it might constitute a possible mechanism contributing to neurorestoration in PD. Pericytes are known to possess certain features of mesenchymal stem cells (MSC) when isolated and expanded in vitro with respect to marker expression and differentiation capacities (Crisan et al. 2008; Guimaraes-Camboa et al. 2017). Evidence is currently accumulating that the secretome of pericytes is stimulus-dependent and might have pro-regenerative properties similar to the ones that are well known for the MSCs secretome (Gaceb and Paul 2018).

In the present study, we described an in vitro model of partial DA lesion using differentiated LUHMES treated with MPP^+^ or 6-OHDA. This model will allow screening of neuroprotective and neuroregenerative substances that especially affect the DA phenotype. Brain pericytes may constitute a novel target for neurorestoration due to their secretory capacities. The secretome of brain pericytes mediated via PDGF-BB/PDGFRβ may represent a possible mechanism contributing to neurorestoration in PD.

## Electronic Supplementary Material

ESM 1 (PDF 2108 kb)
