# Supplementary material for: An In Vitro Partial Lesion Model of Differentiated Human Mesencephalic Neurons: Effect of Pericyte Secretome on Phenotypic Markers
Source: J Mol Neurosci. 2020 May 29;70(11):1914–25. doi: 10.1007/s12031-020-01589-6 (PMC7561585; doi:10.1007/s12031-020-01589-6)
Supplement: Supplementary file 1 — (PDF 2108 kb) [file 12031_2020_1589_MOESM1_ESM.pdf]

An *in vitro* partial lesion model of differentiated human mesencephalic neurons: effect of pericyte secretome on phenotypic markers

Abderahim Gaceb[1], Marco Barbariga[1] and Gesine Paul [1,2].

1 Translational Neurology Group, Department of Clinical Science, Wallenberg Neuroscience Center and Wallenberg Center for Molecular Medicine, Lund University, 22184 Lund, Sweden

2 Department of Neurology, Scania University Hospital, 22185 Lund, Sweden

Corresponding author: Abderahim Gaceb

Address: Translational Neurology Group, Department of Clinical Science, Lund University, Sölvegatan 19, 22184 Lund, Sweden.

E-mail address: abderahim.gaceb@med.lu.se

Journal of Molecular Neuroscience

Supplementary Figure 1

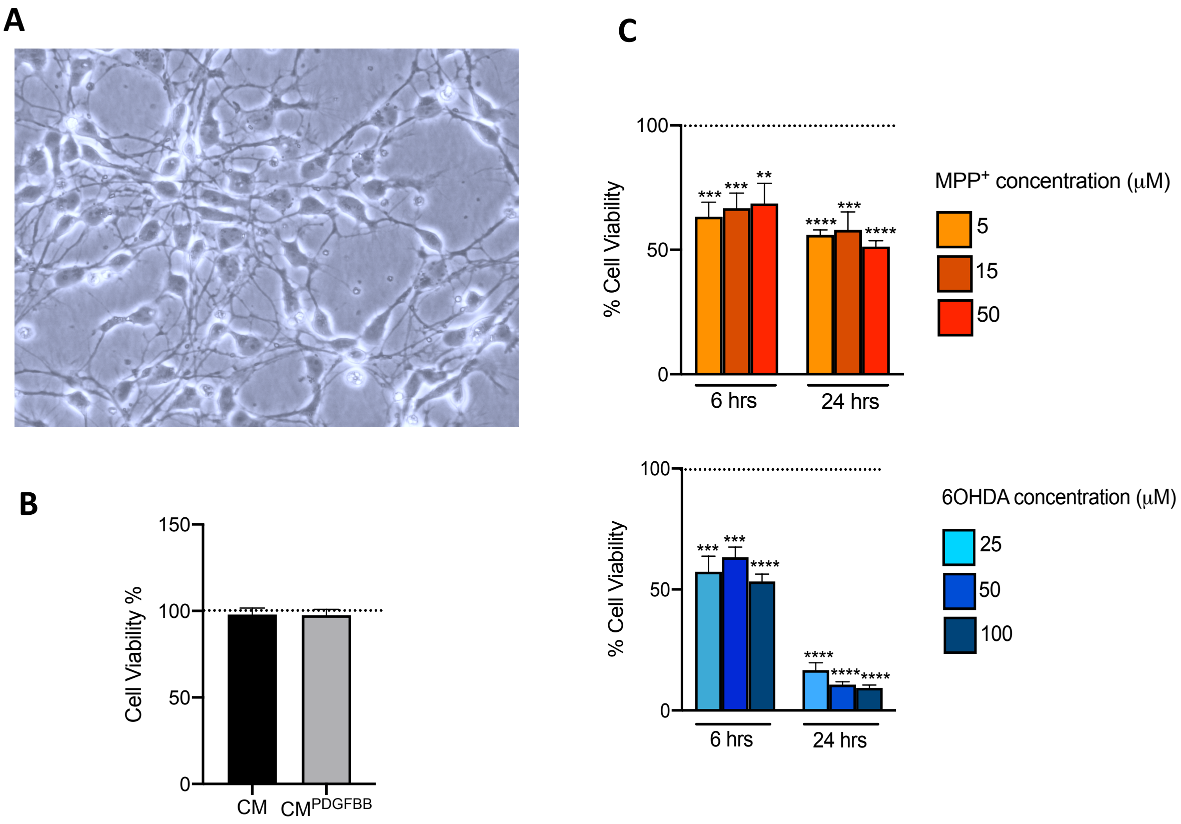

(A) Phase-contrast image (20X) illustrating the morphology of differentiated LUHMES cells (mature neurons).

(B) Effect of pericyte-conditioned medium on DA cell viability using trypan blue. Differentiated LUHMES cells were incubated with non-treated pericytes conditioned medium (CM); conditioned medium of PDGF-BB treated pericytes (CM^{PDGFBB}) for 24hours. Differentiation LUHMES medium was used as a control. The results are expressed as percentage of cell viability and represent the mean ± SD (n=3 independent experiments, each in triplicates).

(C) Trypan blue dye exclusion assay for MPP^+ and 6-OHDA toxins titration. Differentiation LUHMES medium was used as a control. The results are expressed as percentage of cell viability and represent the mean ± SD (3 independent experiments, 3 replicates) ****, $p<0.0001$; ***, $p<0.001$; **, $p<0.01$.
